# Feasibility and uptake of a digital mental health intervention for depression among Lebanese and Syrian displaced people in Lebanon: a qualitative study

**DOI:** 10.3389/fpubh.2023.1293187

**Published:** 2024-01-22

**Authors:** Jinane Abi Ramia, Racha Abi Hana, Philip Noun, Pim Cuijpers, Kenneth Carswell, Edith van't Hof, Eva Heim, Edwina Zoghbi, Marit Sijbrandij, Rabih El Chammay

**Affiliations:** ^1^Department of Clinical, Neuro and Developmental Psychology, World Health Organization (WHO) Collaborating Center for Research and Dissemination of Psychological Interventions, Amsterdam Public Health Research Institute, Vrije Universiteit Amsterdam, Amsterdam, Netherlands; ^2^National Mental Health Programme, Ministry of Public Health of Lebanon, Beirut, Lebanon; ^3^International Institute for Psychotherapy, Babeş-Bolyai University, Cluj-Napoca, Romania; ^4^Department of Mental Health and Substance Use, World Health Organization, Geneva, Switzerland; ^5^Institute of Psychology, University of Lausanne, Lausanne, Switzerland; ^6^Department of Psychology, University of Zurich, Zurich, Switzerland; ^7^Country Office for Lebanon, World Health Organization, Beirut, Lebanon; ^8^Psychiatry Department, Faculty of Medicine, Saint Joseph University, Beirut, Lebanon

**Keywords:** digital interventions, depression, low-to-middle income countries, displaced people, dropout, uptake, qualitative evaluation, Step-by-Step

## Abstract

**Introduction:**

Digital interventions are increasingly regarded as a potential solution for the inaccessibility of mental health treatment across low-and-middle-income settings, especially for common mental disorders. Step-by-Step (SbS) is a digital, guided self-help intervention for depression found effective in two Randomized Controlled Trials (RCTs) in Lebanon. For research implementation and further scale-up, this paper reports the results of a qualitative evaluation of SbS among the Lebanese and others and displaced Syrians in Lebanon.

**Methods:**

Thirty-four Key Informant Interviews (KIIs) were executed with participants of the RCTs, SbS staff members, and external stakeholders. Questions garnered feedback about the feasibility, acceptability, enabling factors, and barriers to adhering to the research, implementation, and the SbS intervention. A thematic analysis was conducted using NVivo, and key themes, topics, and recommendations, on research methods and the intervention itself, were generated and reported.

**Results:**

Results showed a high level of acceptability of SbS among Lebanese and Syrians and identified sub-groups for whom acceptance or use might be lower, such as older adults and people with limited access to the internet or smartphones. Furthermore, interviews identified the main enabling factors and barriers to adherence related to the research design, content, and delivery approach. Barriers related to feasibility included lengthy assessments as part of the RCTs, and mistrust related to delays in study compensations. Other common challenges were forgetting login credentials, poor internet connection, being busy and competing needs. Enabling factors and best practices included motivating participants to use the intervention through the weekly support provided by helpers, setting an oral contract for commitment, and dividing the compensations into several installments as part of the RCTs. Recommendations regarding sustainability were given.

**Discussion:**

The findings show that overall, SbS is feasible, acceptable, and much needed in Lebanon among the Lebanese and Syrians. This assessment identifies reasons for low adherence to the research and the intervention and presents improvement solutions. Recommendations generated in this paper inform the upscale of SbS and the planning, design, and implementation of future digital interventions in research and service provision settings in the mental health field.

## Introduction

Digital mental health interventions are increasingly regarded as a solution to the global inaccessibility of mental health treatment (1). They present an accessible, scalable, and practical medium for care delivery at a low cost (2). The burden of disease associated with mental health conditions is highest in Low and Middle-Income Countries (LMICs) where previous estimates suggest a large mental health treatment gap of ~75% among adults (3). LMICs could benefit from digital mental health interventions, considering the high demand and the widespread use of smartphones and the Internet (1, 4, 5).

Strong evidence exists around the effectiveness and cost-effectiveness of digital mental health interventions (6–9). Nevertheless, there is a dichotomy between the promising evidence for these innovations and the low uptake observed among users (2, 10). Challenges to their uptake are often observed during the research phases, including low adherence to treatment among research participants, decreased completion rates at the post-assessments, and high dropout rates during research trials (11, 12). Meta-analyses showed that the average attrition rate was 57% for computerized mental health interventions; non-adherence, including dropout from the treatment or non-completion of assessments, ranged from 28% for therapist-guided digital interventions to 74% for unguided interventions (13). This high attrition could risk underpowered studies, affect the validity of the effectiveness studies, or it might indicate a low uptake in the community upon scale-up (11, 14, 15).

A few implementation studies investigated the reasons for attrition and low adherence to digital interventions in high-income settings. Reasons were categorized into intervention-related and user-related. Intervention-related reasons included poor usability of the intervention, cultural irrelevance of the content, bugginess, limited usefulness in emergencies, and time required to sign up and enter data. User-related reasons were a lack of motivation associated with depression, lack of trust or perceived benefit of the digital treatment, security and privacy concerns, losing interest, and low health literacy (2, 11). Nonetheless, little is known about the factors that may promote the uptake of digital interventions in research and real-life implementation and integration into existing health systems (3, 16). Furthermore, little is known about their uptake in LMICs and the reasons and solutions for dropout among populations affected by adversities (3); hence the need for implementation and attrition studies to understand the barriers to adherence and enabling factors for the uptake of digital mental health interventions in LMICs.

The World Health Organization (WHO) has developed a set of scalable interventions to address common mental disorders. One of these interventions is Step-by-Step (SbS), a 5-week guided self-help intervention for adults experiencing depression. It includes a narrated story, a set of techniques and exercises to reduce depression symptoms, with minimal remote support by trained non-specialists called “e-helpers” (17, 18). SbS was developed, culturally adapted, and pilot tested by WHO and the National Mental Health Programme (NMHP) in Lebanon among the Lebanese population and displaced Syrians (19–21). A feasibility randomized controlled trial (RCT) (22) followed by two fully-powered RCTs assessed its effectiveness and cost-effectiveness in the local setting (23, 24). The intervention group had access to the SbS intervention and weekly e-helper support. The Enhanced Care As Usual (ECAU) group received access to one page of psychoeducation on depression and anxiety, a referral list to primary healthcare centers, and the national lifeline for emotional support and suicide prevention (21). Quantitative results showed that the SbS intervention is an effective and cost-effective treatment for depressive symptoms, functional impairment, and anxiety. These results informed the decision to scale it up into a national service in Lebanon; The average dropout rates reported during the RCTs at post-assessments were high, 46.2% for Syrians and 65.1% for Lebanese (23, 24). Hence the need to investigate the reasons and solutions for the high attrition encountered during the trials.

Lebanon is a middle-income country with political turmoil and a fragmented healthcare system further strained by the influx of more than 1.5 million Syrian displaced people in the past 10 years (25). In 2006, the gap in mental health treatment was ~90% (26, 27). Since 2020, and in conjunction with the study implementation, the country has been struggling with a humanitarian emergency caused by severe political, economic, and financial problems. Additionally, COVID-19 regulations and the widespread street protests that erupted in 2019 further exacerbated the situation. In August 2020, an explosion at the Port of Beirut killed more than 200 persons, injured thousands, and critically damaged the healthcare sector, according to WHO reports (28), The systemic failures and the multilayer crises deteriorated the mental wellbeing of the population to a great extent and increased the need for nationwide mental healthcare interventions.

In this qualitative study, we aimed to assess the acceptability and feasibility of using SbS in Lebanon and investigate the reasons and solutions for dropout from the lens of the users, staff, and stakeholders. We looked into the challenges faced during the research trial, the best practices, and the recommendations for scale-up. They were examined from a multilevel perspective: content-wise, delivery approach, research methods, context-related factors, and scale-up plan. Throughout this evaluation, we aimed to answer the following research questions: (1) Is SbS considered acceptable, relevant, and beneficial among Lebanese and Syrian populations in Lebanon? (2) What are the promoting and hindering factors that affect the success of the SbS research and intervention uptake in Lebanon? (3) What are the challenges and recommended modalities for sustainability in Lebanon?

Findings should inform the upscale of the intervention into a national service beyond the scope of the research and guide the design and implementation of similar interventions worldwide.

## Methods

### The intervention

Step-by-Step is a brief 5-week digital, guided, self-help intervention for adults with depression, delivered through an application or a website, with a minimal 15-min a week of remote guidance provided by trained non-specialists called e-helpers (18, 29). SbS comprises core strategies, behavioral activation, stress management, problem management, increasing social support, and relapse prevention techniques. The techniques are delivered through narrated story-based weekly sessions and practical exercises, which are audio recorded and available in English and Arabic. Participants get to practice activities between the sessions, such as grounding and slow breathing exercises, scheduling activities using an online calendar, a gratitude list exercise, a mood tracker, and simple self-care, while more complex activities should be split into smaller steps. They receive brief, maximum 15-min weekly calls, or messages from e-helpers. E-helpers are supervised non-specialists trained to provide emotional support and motivation throughout the program (18). E-helpers follow preset support templates, guides, and protocols to deliver their service. In an introductory call, they set an “oral contract” with participants to motivate them to respond to their contacts and to commit to the sessions and activities to maximize their benefits. E-helpers then follow up with them weekly, as per the agreed method of contact (phone call or message support).

SbS was tested and delivered by NMHP at the Ministry of Public Health in Lebanon. One thousand two hundred and forty-nine participants were recruited through social media and outreach methods and were included in the study upon completing online self-assessments and scoring above the cut of score on depression and functioning (37, 38). Recruitment took place between December 2019 and June 2020.

### Data collection and procedures

We conducted 34 Key Informant Interviews (KIIs) with study participants, SbS staff, and external stakeholders between September 2020 and January 2021. Out of the 34 KIIs, we held 14 KIIs with study participants in the intervention group who were selected following a stratified random sampling method. All participants were asked for consent to participate in the interviews by their corresponding e-helpers upon completion or dropout (Supplementary material 1). We then stratified participants by gender (male/female), nationality (Syrian/Lebanese and other populations residing in Lebanon), completion status (completer/drop-out), and preferred support method (message/call). It is noteworthy that 14 participants from the ECAU group were interviewed to understand about their perspective on the research and potential reasons for dropout or motivators for adherence, yet were not reported in this paper as the main focus is on the intervention adherence. Using the SPSS software, we automatically generated a random sub-sample of individuals for every variable above, and we reached out to the participants identified for an interview. All seven SbS staff members, five e-helpers, the project coordinator, and the clinical supervisor (both referred to as supervisors in the results for confidentiality), were included as key informants. For the external stakeholder group, we selected 13 key informants by convenience. These were partnering with non-governmental organizations (NGOs), project counterparts from NMHP and WHO, and outreach Syrian volunteers, who were part of the project's steering committee or helped disseminate SbS among their networks. The steering committee members contributed to designing and planning the RCTs and the SbS content through binary meetings. The e-helpers contacted the external stakeholders by phone, email, or chat, and received their consent to be contacted and interviewed. Table 1A details the distribution of study interviewees across genders, age, marital status, and nationalities. Table 1B details the information on the steering committee stakeholders interviewed by organization and position. Organizations and positions were not specified in the results to maintain confidentiality, rather referred to as external stakeholders.

**Table 1A:** Overview of key informant study participants form the intervention group stratified by different stakeholder groups, study groups, completion status, nationality, and sex.

	**SbS Intervention group participants interviewed (n** = **14)**	
	**Completed SbS (*****n*** = **10)**	**Dropped out SbS (*****n*** = **4)**
Age, *M* (SD)	Mean 30.1 (7.47)	Mean 34.2 (10.78)
Sex, *n* (%)	Male = 3 (30%) Female = 7 (70%)	Male = 2 (50%) Female = 2 (50%)
Marital Status, *n* (%)	Single = 3 (30%) Married = 7 (70%)	Single = 2 (50%) Married = 2 (50%)
Nationality, *n* (%)	Lebanese = 2 (20%) Syrian = 8 (80%)	Lebanese = 1 (25%) Syrian = 3 (74%)

Dropout: study dropouts who either proactively asked to be dropped out or discontinued and did not complete the post-assessments. It's noteworthy that all the dropouts interviewed were also intervention dropouts and didn't complete the sessions.

**Table 1B:** Overview of key informants stratified by different stakeholder groups, positions, and sex.

	**SbS project staff and external stakeholders interviewed (*****n*** = **20)**	
	**SbS project staff (*****n*** = **7)**	**External stakeholders (*****n*** = **13)**
Sex, *n* (%)	Male = 2 (29%) Female = 5 (71%)	Male = 3 (30%) Female = 10 (70%)
Organization	National mental health programme	National mental health programme United Nations high commissioner for refugees World Health Organization Lebanon ACTED Lebanon NGO International medical corps ABAAD - Resource Center for gender equality Syrian facebook group
Position	Project coordinator Clinical supervisor E-helper	Head of program Found and director Operations manager Service development coordinator Advocacy and policy coordinator Protection advisor Protection officer National officer for noncommunicable diseases and mental health Mental health coordinator Facebook group admin

The project coordinator and clinical supervisor conducted in-depth interviews with the participants. An independent researcher (co-author), not part of the SbS management team or the original research trials, interviewed the study team to limit bias, as e-helpers interviewed the external stakeholders. All interviewers received a 1-h training on the semi-structured interview guides with open-ended questions. Interviews with SbS staff were conducted face-to-face, while those with participants and external stakeholders took place over the phone. Interviews lasted 45 min on average, all interviews were audio-recorded. The project coordinator and the independent researcher transcribed and translated them into English.

For stakeholders, a semi-structured interview guide with 13 open-ended questions explored their general feedback on SbS, the degree of acceptability and relevance of SbS to the different cultural groups in Lebanon, and its ease and feasibility. Furthermore, the guide explored unforeseen implementation challenges and recommendations for future integration into the healthcare system in Lebanon (Supplementary material 2).

For project staff, the guide consisted of 16 questions. They explored their general experience with SbS, rapport with participants, observed reasons for attrition and solutions to improve adherence, and recommendations on managing their workload, management system, supervision modality, and retention (Supplementary material 3).

The interview guide for study participants included 20 questions that examined their overall impressions on participating in the SbS research study, using a digital mental health intervention, and its perceived benefits. Questions also assessed SbS's feasibility and acceptability in Lebanon. Additionally, feedback and suggestions for improvement were collected on the content of the intervention, the rapport with the e-helpers, the registration process and assessments, and barriers and enablers for adherence to the study and the intervention (Supplementary material 4).

**Figure 1:**
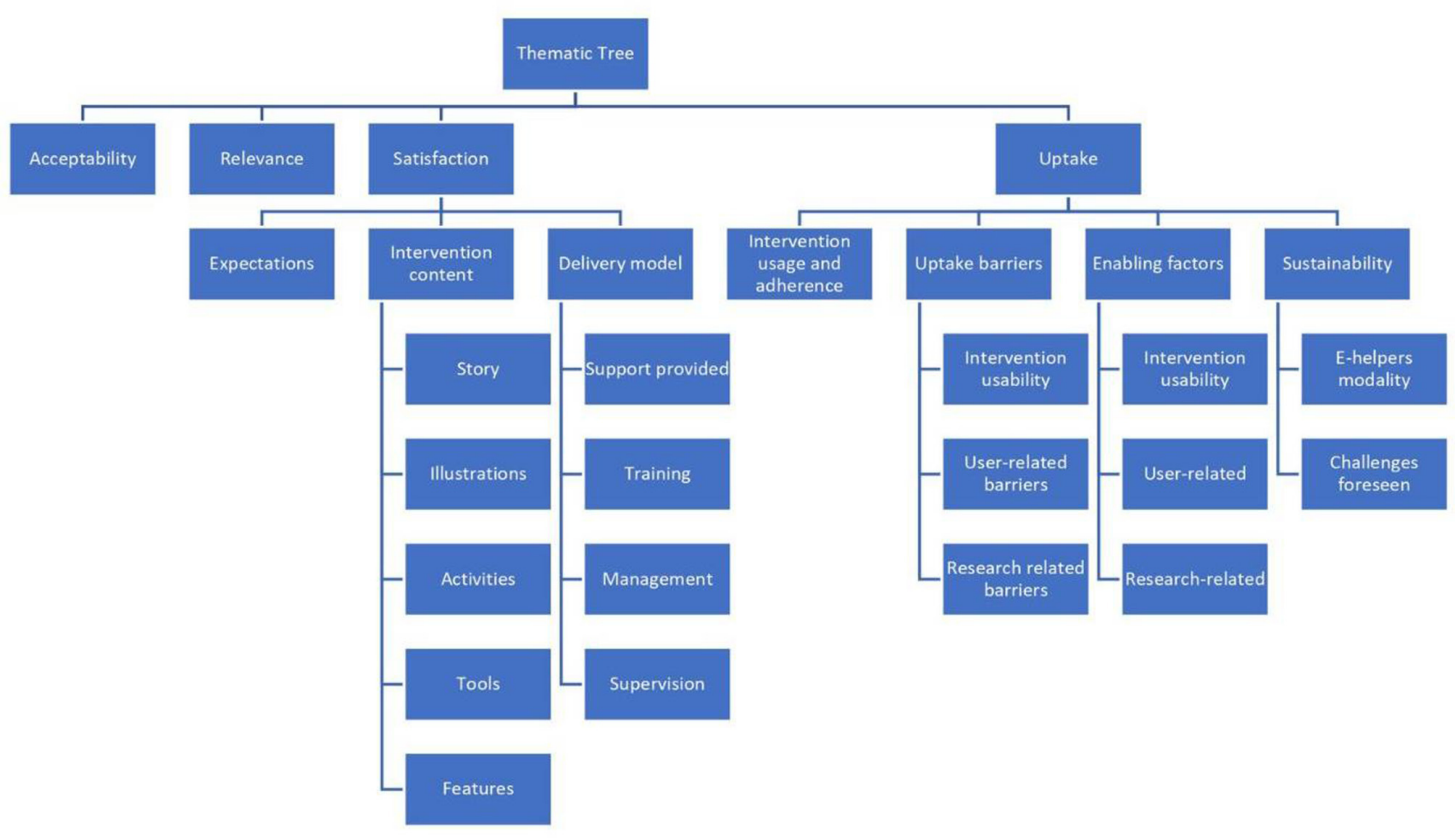
Thematic tree of the Step-by-Step qualitative evaluation analysis.

The first author conducted a thematic analysis of the transcripts using Nvivo 2017, following the framework approach. This approach consisted of following pre-set themes that were generated from the interview guides, familiarizing oneself with the interview transcripts, and generating emerging codes under each theme (39). An independent researcher (co-author) who had conducted interviews with e-helpers then carried out random checking of the coding on a sample of the interviews and confirmed the validity of the codes and the thematic tree created. Key informant sub-groups were analyzed separately, data was triangulated to report commonalities and divergences in responses, and the final results were cross-checked between the two researchers. The participant subgroups were analyzed individually, before triangulating the data across groups to identify common themes and compare between the different groups. Figure 1 presents the thematic tree generated following the qualitative analysis.

## Results

### Acceptability

Acceptability was defined as the extent to which people would accept taking part in a research project, receiving online treatment, and committing to the notion of self-help for improvement. Overall, there was consensus among the 34 key informants that the SbS intervention was much needed, beneficial, practical, and culturally relevant for the different populations in Lebanon (Lebanese, Syrians, and other populations residing in Lebanon). Many found it innovative in the Arab context, especially considering the global shift toward online treatment post-COVID-19 pandemic. At first, three staff members displayed skepticism about the acceptability of this self-help digital intervention in Lebanon. Nevertheless, it became evident to them throughout the research that people accepted and benefited from the service. All seven staff members were confident that the SbS project could be well-received in Lebanon. They listed several promoting factors for the acceptability such as SbS provides an affordable and practical solution to the increasing demand for mental health services, there's a general acknowledgment and normalization of mental health, which enhances the help-seeking behavior of those in need, people are more sensitized and used to internet interventions, especially after COVID-19 pandemic, which makes it easier for them to accept the self-help digital intervention, and SbS ensures the confidentiality and privacy of users amidst the prevailing stigma around mental health. This last point was highlighted by the study whereby stigma was reported as a prevailing barrier among key informants; almost half of the study participants interviewed didn't disclose to anyone that they participated in SbS due to fear of being stigmatized.

### Relevance to the target population

**Figure 2:**
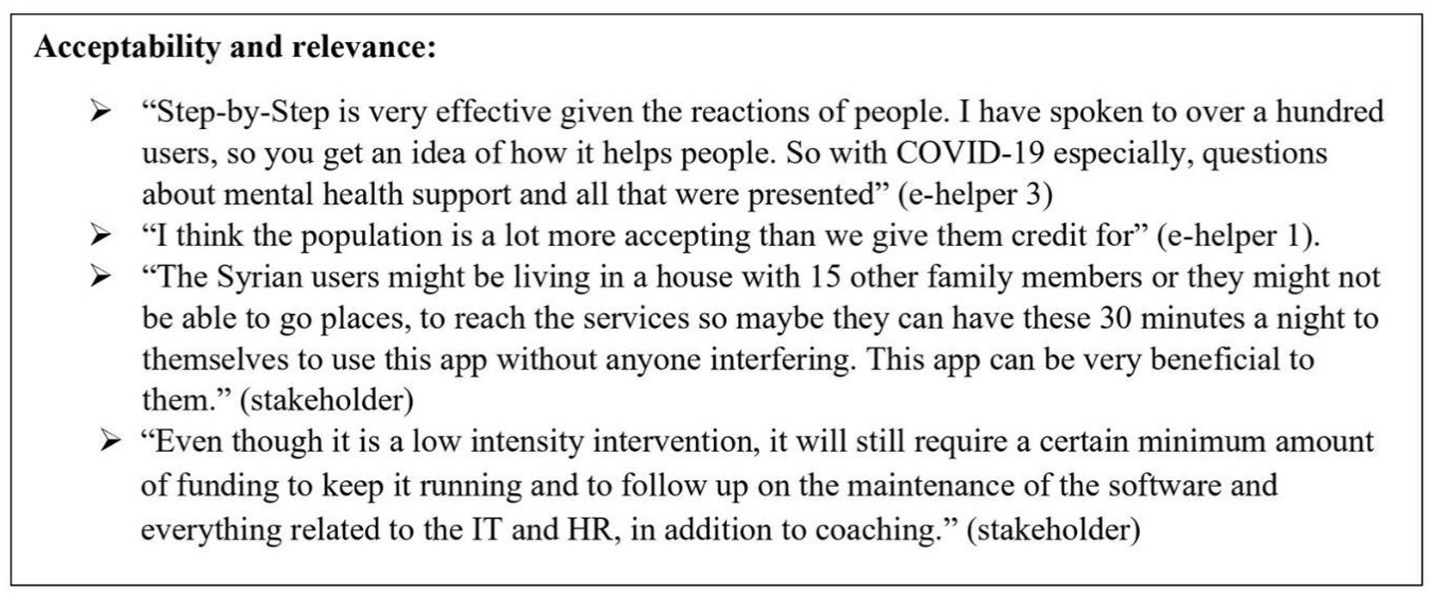
Quotes from key informants on the acceptability and relevance of Step-by-Step in Lebanon.

It was generally believed among all respondents that all cultural groups could relate to SbS and find it acceptable for them to use a digital mental health intervention. Yet, external stakeholders specified different levels of acceptability among population groups. They noted that young adults and the tech-savvy would be more accepting of SbS than the rest. Additionally, some external stakeholders thought that displaced Syrians would accept SbS more than the Lebanese because the former are more exposed to mental health awareness and services through the humanitarian NGOs working in the field. On the other hand, four Syrian external stakeholders noted that it might take some time before the displaced populations accept such interventions delivered by the Ministry of Public Health due to the mistrust in applications and the government's intentions and agenda. Two external stakeholders believed that SbS would not be relevant to the older adults, people with low technological literacy, displaced populations with low literacy levels, migrant workers due to language barriers, people having intellectual disabilities, and people with impaired vision or hearing. Only one external stakeholder was skeptical about its uptake among people with depression because people living in difficult and worsening situations would be looking for solutions whereas the program did not offer solutions but rather techniques to cope with difficult symptoms. Figure 2 summarizes quotes on the acceptability, and relevance of SbS.

### Satisfaction

#### Expectations

All 14 key informants in the participants' intervention group were satisfied with SbS and conveyed that it was up to or exceeded their expectations. Some revealed that they didn't have any expectations or thought at first it was spam but were then amazed by the benefits of this program. Most key informants valued confidentiality, credibility, and e-helpers' support. Only a few thought that face-to-face support would be more effective for some, while one was concerned that SbS might not be helpful due to the severity of the contextual problems experienced that highly impact the severity of the depressive symptoms. When asked about the underlying reasons for signing up for the SbS program, study participants' responses included shock experienced following the Beirut explosion, struggling with the deteriorating financial situation and unemployment, lack of services available, fear of contracting COVID-19 in face-to-face support, divorce, and rape by their husband.

#### Intervention content

##### Story and illustrations

All key informant study participants in the intervention group liked the story and related to the characters and the realism of the events and symptoms. Most of them appreciated being able to select their preferred character and storyline. The cultural relevance of the characters, illustrations, and storylines was highly praised by most respondents. E-helpers also confirmed that their users conveyed, during support sessions, that they benefited from and enjoyed the story content and illustrations and found them very relatable.

Several areas of improvement were identified by the e-helpers and participants. Two e-helpers considered the illustrations to be a bit childish although no negative comments were received from the participants on this matter. E-helpers also felt that repetitiveness in the storyline would be a barrier to adherence to the program, especially for people with depression. One e-helper felt that the Syrian users had more serious problems than the story could offer. For example, one Syrian female participant couldn't relate to the social support exercise as she expressed that women in Arab culture are taught to keep their feelings to themselves and not reach out for help or cry in front of anyone. Another Syrian participant felt ashamed to share her feelings but was relieved to find them in the story.

Two e-helpers mentioned that users preferred and applied the sessions that included fewer and simple exercises, while those that included multiple exercises and more complex and social activities were reported to be overwhelming, especially to those with social anxiety.

##### Activities and tools

Most of the study participants interviewed and all e-helpers conveyed that their favorite exercise was “slow breathing” because it was easy to implement and had direct and tangible benefits on users. Participants reported that they practiced the breathing exercise before sleeping to help relieve their anxiety and insomnia, while others used it to calm down when distressed upon encountering a stressful event. Both males and females resorted to it to manage their stress and anger during COVID-19 quarantine times. One participant voiced that the exercise was difficult to apply at first, but with practice, it became easier.

The second most preferred feature, according to most key informant participants, was the “mood tracker,” which was described as a friendly way to track and notice their feelings and mood. Most users adhered to it regularly after receiving the push notifications or even without any reminders. They noted down all their moods by selecting the relevant emojis, whether happy, sad, or angry, while a few said that they didn't use it when feeling down or angry.

Among other preferred activities were the “small self-care activities” exercise (e.g., walking, drinking tea, listening to music, etc…), the “gratitude list” (list of the things in life one is grateful for), and “the positive self-talk exercise” (being kind to oneself and avoiding self-blame through encouraging words). The benefits of these activities were sensed by some participants only after finishing the program, or upon being subjected to negative talk by their surroundings. Other study participants reported benefiting from the “listing the warning signs” activity, especially among those who experienced burnout in their jobs. Only one key informant participant mentioned that he didn't use the gratitude list because he didn't understand what to write in it.

The exercise for helping the management of more challenging regular tasks, which consisted of dividing a “big task” into small steps and planning and scheduling them in a calendar, received mixed reviews from key informant study participants. Some revealed that they learned how to divide big tasks into smaller steps and plan for them ahead of time, whereas others found it difficult to apply. Participants who used to be active or had a hobby in the past found it easier to regain their activity than those who had to integrate a whole new activity into their daily life. Suggested complex activities were thought to be overwhelming and time-consuming or not feasible during COVID-19 times as most of them entailed outdoor activities. Nonetheless, some users valued their importance once they had finished the program.

##### Features

**Figure 3:**
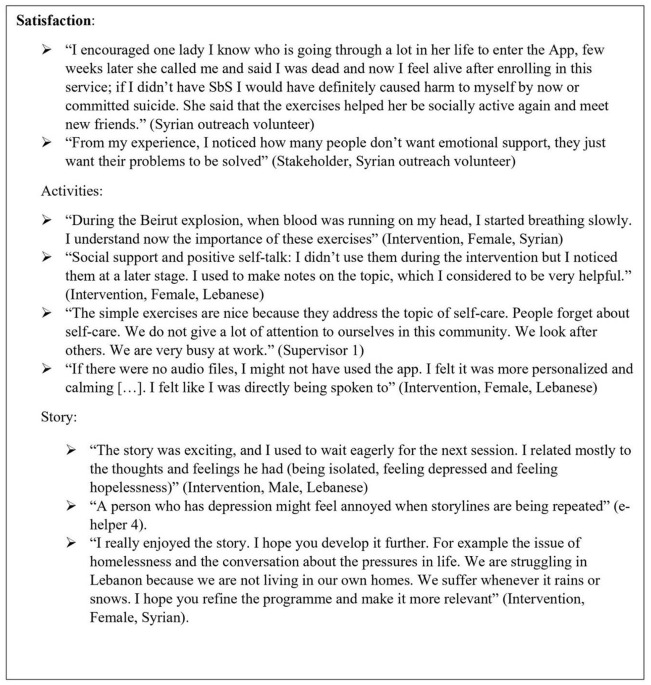
Quotes from key informants on satisfaction with Step-by-Step.

The audio recording feature of the intervention (the application content was fully audio recorded) was considered an important feature by almost two-thirds of the key informant study participants who used it. They reported that the recordings helped them stay focused, engaged, and interested in the program, they calmed them down and made them feel that the intervention was more personalized and humane, and they were very convenient for those who couldn't or didn't like to read. Among those who didn't use the audio feature, the common reasons reported were privacy concerns and not wanting anyone to listen, a matter of preference and better focus when reading, or because they assumed it would be a voice of a robot and not of a real person. Figure 3 reports quotes on satisfaction with Step-by-Step.

### Delivery model

#### Support provided

All 14 key informant participants from the intervention group appreciated the support provided by the e-helpers and considered it an important motivation for them to complete the program. The majority mentioned that they wouldn't have benefited as much from the intervention without the e-helper support. This was validated by most of the staff. Participants especially valued the great listening skills, the problem-solving approach, the safe space provided, and the punctuality of e-helpers. All key informant participants in the intervention group thought that SbS wouldn't work properly without the e-helpers' support because it would be too impersonal. According to one of the supervisors, the specificity of the culture in Lebanon promotes the e-helpers' role because people need to talk to someone about their problems and this is by far their preferred component. Overall, key informant participants were satisfied with the time and frequency of the support which was 15 min per week.

#### E-helpers' training, management, and supervision

E-helpers viewed the 5-day training received at the beginning as very beneficial yet condensed. Nevertheless, they reported that the training content about depression and the therapeutic approaches was found to be generic and not tailored enough to the realities of the local context. The preparatory phase, in the beginning, consisted of ~300 h (1½ months) of on-the-job role plays, trained to use the protocols and the support templates. It was reported to be crucial in equipping them with the knowledge and skills needed to provide support. Work protocols were comprehensive yet lacked a section about implementation changes and potential risks encountered in the local context.

As for the caseload and work modality, e-helpers conveyed that their workload fluctuated starting with peaks followed by low periods, based on recruitment rates of participants. They considered the 4-h shift a fair amount of time to support six participants instead of eight, as originally designed. E-helpers reported that on average they needed more than the allocated 30 min to support every person and write the case notes, and to have some spare time to prepare for their support contacts and conduct administrative tasks. Additionally, e-helpers preferred to work from the office instead of from home during the COVID-19 pandemic because they benefited from face-to-face peer support and knowledge exchange. They felt a sense of belonging which increased their motivation to work. As for the duration of the calls, it was noted that at the beginning, calls and messages took much longer than 15 min (~30 min to 1 h). Nonetheless, with training and further refining of the support messages templates, e-helpers were able to stick to the time range. Everyone agreed that 15-min a week was very adequate for this type of support.

With regards to management and supervision, e-helpers reported that the weekly group supervisions were very beneficial whereby they allowed information exchange with the circular round of feedback. Two e-helpers suggested that the supervision could be improved by in-depth discussions of one or two cases instead of covering all cases during every meeting. They also suggested getting optional individual supervision occasionally. The two supervisors mentioned that fidelity checks, weekly meetings, one-on-one calls, and reviewing case notes and messages were all very important for quality assurance and performance improvement. Similarly, the very safe and open work environment fostered a constructive learning space for team members. For the supervisors, the main challenge encountered, in quality assurance and conducting fidelity checks, was when it was done remotely over the phone during the COVID-19 pandemic (when e-helpers worked from home). Several challenges impeded the proper supervision of the calls such as internet problems and time conflicts between the clinical supervisor and the e-helpers' contact sessions.

### Uptake

#### Intervention usage and adherence

Most of the key informant participants in the intervention group disclosed that they had used SbS either every day or two to three times per week at the most. Participants mostly used it at night before sleeping or in the morning upon receiving a notification to input their mood. Two participants mentioned that they opened the app whenever they felt upset. One participant revealed that she didn't use SbS much at first but then when she noticed her improvement, she started using it more often, around three times a week. Another person still revisited the story after completing the program while all the other informants mentioned that they still applied the “slow breathing exercise,” the simple “self-help activities,” and the “behavioral activation techniques” in their everyday life.

#### Barriers of adherence

##### Intervention usability

The most common issues raised by the study participants, confirmed by e-helpers, were struggling with the slow internet, difficulty in setting their username and password, forgetting their passwords after logging out, the two-step authentication, lack of space to download the app, forgetting to log in upon turning off notifications in the app, changing phones, forgetting how to download the app (mostly among Syrians), and having old phones (which caused problems with the SbS software). E-helpers also mentioned that the technical issues and bugs faced at the beginning of the trial were impeding their work. They reported that it was very time-consuming for them to test and report these bugs, and to tailor their support templates to clarify all this to the users. One e-helper mentioned that some population groups encountered more difficulties while using the app than others, namely the Syrian population and older adults. E-helpers found that the back-end office of the platform was not very easy to use, and two staff members were concerned that the app would be easily outdated as compared to apps developed in the private sector. E-helpers also noticed that users who chose message support became more inactive and unresponsive as compared to those who opted for phone calls. This was validated by one participant who stressed the importance of phone calls for motivation, engagement, and effectiveness of support compared to messages.

##### User-related barriers

Among the intervention group, the vast majority of the key informants as well as the e-helpers mentioned that they dropped out because of their busy lifestyle. One user who completed SbS revealed that she was about to drop out but she motivated herself to continue, while another speculated that some people might just feel better and discontinue for that reason. Staff members considered the main inhibitor to users' adherence was signing up for the wrong motives such as getting compensation instead of getting treatment.

#### Enabling factors

##### Intervention design and engagement

Among the main enabling factors for using SbS mentioned by key informant participants in the intervention group, were the simplicity and user-friendliness of the app/web design, the soothing and relaxing colors, the diversity of the characters that users could relate to and identify with, and the very fast response to any technical problems encountered. Furthermore, the engagement through push notifications and the mood tracker served as reminders according to most intervention group key informants. Most Lebanese participants and staff mentioned that the calls and WhatsApp message reminders from e-helpers helped them to adhere to the intervention. Another factor mentioned by the staff was the “oral contract” that was set between the e-helpers and the intervention group participants, where the latter pledged to commit to the program and not exceed the limited number of contact sessions.

##### User-related factors

A small minority of the respondents, both from the Lebanese and Syrian groups, attributed their retention to their inner motivation and their perceived benefit of the free treatment.

##### Research procedures

The financial compensation for participating in the research was mentioned as a very effective incentive among most Syrian respondents and one Lebanese respondent. Furthermore, most respondents highly valued dividing the compensations into three installments instead of handing them in one bulk at the end of the research, as was done in the previous research phases.

**Figure 4:**
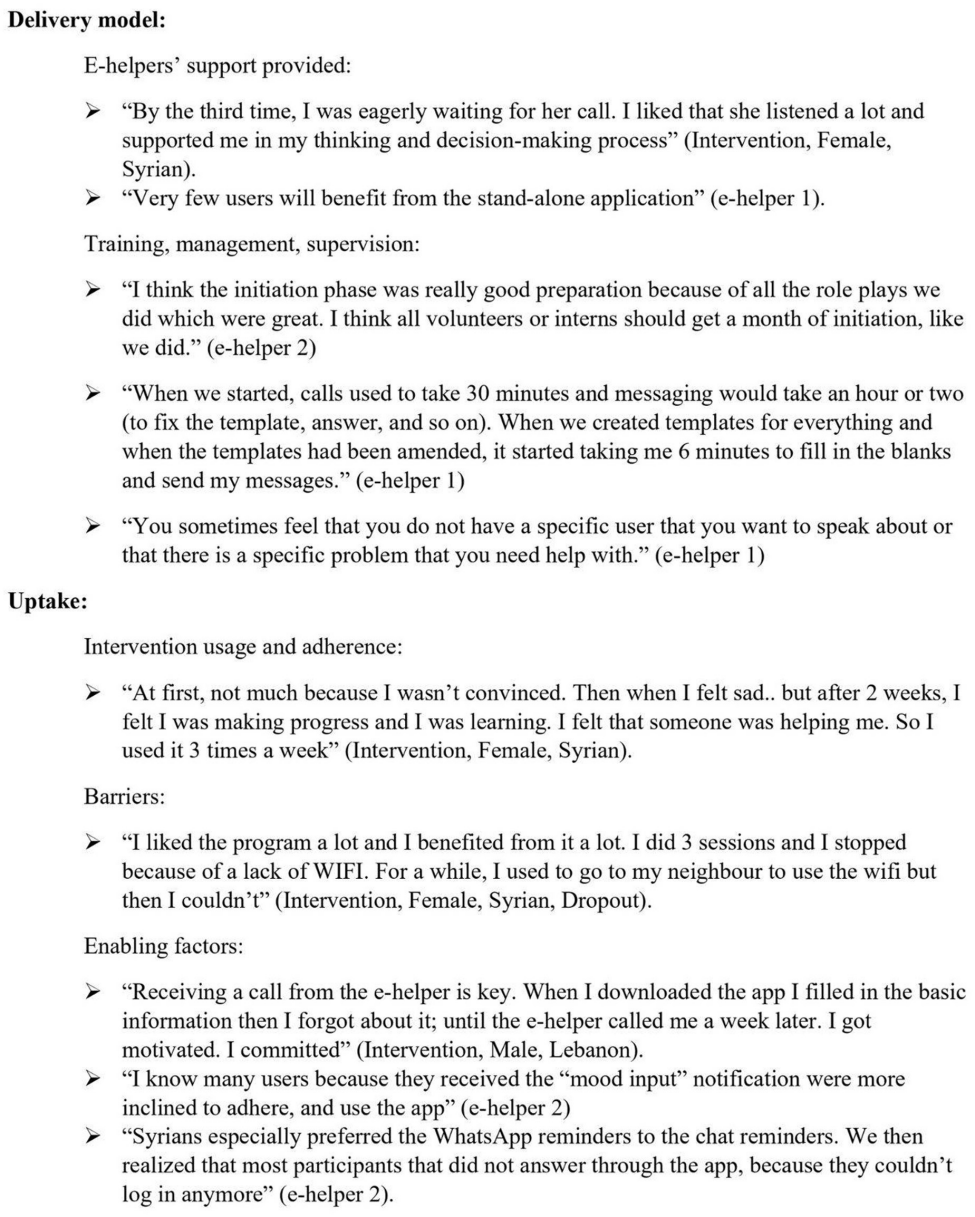
Quotes from key informants on the delivery model and uptake within Step-by-Step.

Figure 4 details quotes about the delivery model and the uptake of Step-by-Step.

### Sustainability of the service

#### E-helpers' modality

Key informant staff and external stakeholders were asked about their perception of the sustainability of the e-helper's modality in the long run, beyond the research setting. About half of the staff members envisioned that the volunteering system could work with the creation of a flexible shift schedule, thorough supervision, a culture to promote humanitarian values, a sense of belonging, and a focus on professional development. A minority of staff thought that a volunteering e-helper model would not be sustainable because it would be too draining for e-helpers and would require commitment. Another small proportion of staff suggested an internship model whereby university psychology students could be e-helpers who complete a certain number of hours in the SbS program. With regards to the internship model, one supervisor raised concerns that interns might be more motivated to fill the required number of hours for their credits than provide support to users and that there might be confusion between clinical work and basic support, especially if the interns are psychology students.

For all external stakeholders, the volunteering model was thought to be problematic because of the high risk of turnover which would jeopardize the continuity of care provided within the SbS service. Also, the e-helpers shift times are within normal working hours which might pose a limitation for the volunteers to find other jobs and hence increase the risk of turnover.

#### Challenges of sustaining Step-by-Step in Lebanon

External stakeholders considered the feasibility of scaling up and sustaining SbS in Lebanon using two different angles: contextual and project-related. Contextually, SbS was deemed feasible and practical to use even in the most remote settings because of the flexibility it allows in terms of time and place of usage. Nevertheless, some mentioned that the difficulty to secure an internet connection or access to smartphones, among people of low economic status or living in rural areas, might pose a barrier to downloading the application, noting that using it doesn't require internet access all the time. Other challenges listed by stakeholders were stigma, lack of awareness about mental health and self-care, the possible resistance to receiving online support, and the competing priorities related to livelihood.

**Figure 5:**
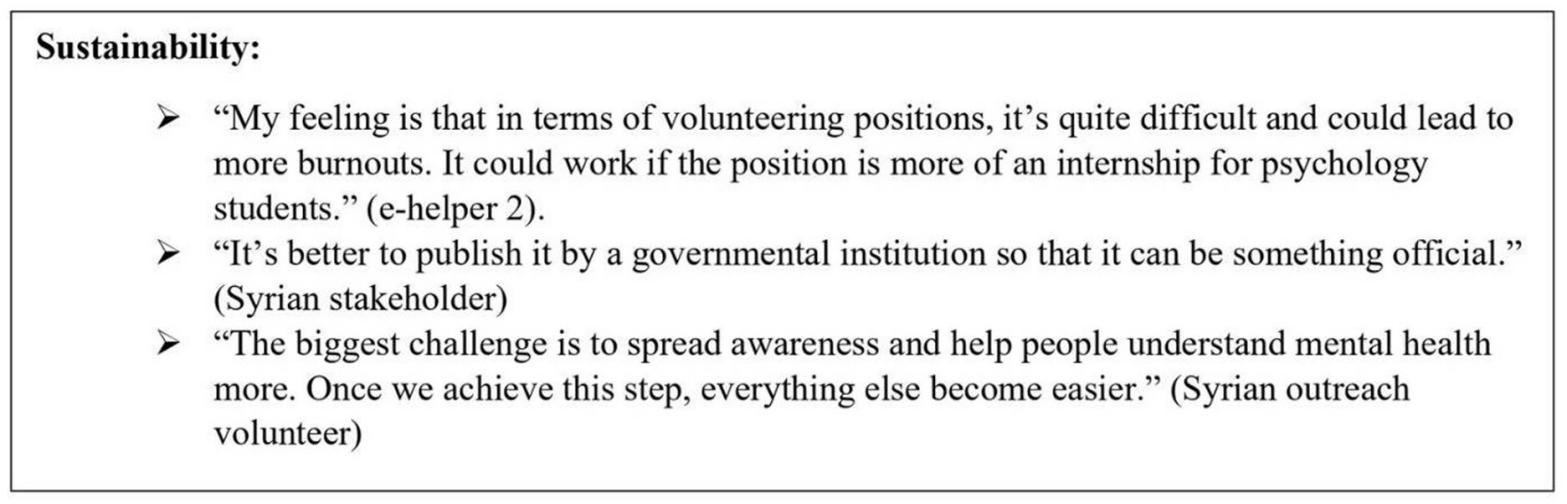
Quotes from key informants on the sustainability of Step-by-Step.

Project-related challenges included difficulty in securing funding to run the intervention in the long run and to sustain the e-helper service with the high turnover expected. Additionally, there was a risk foreseen by some external stakeholders related to the surge of competing self-help programs that would be launched before scaling SbS. Furthermore, Syrian outreach stakeholders raised a concern about the absence of monetary incentives for users upon scale-up which might demotivate people to sign up or complete the intervention. Instead, they proposed focusing on the gained benefits of this intervention. Quotes on the sustainability theme are reported in Figure 5.

## Discussion

This qualitative assessment evaluated the acceptability, relevance, feasibility, and uptake of the digital mental health intervention SbS in an LMIC, Lebanon, among the Lebanese population, and the displaced Syrians. Promoting factors and barriers to the uptake were explored and recommendations of the content, delivery approach, research methods, and implementation beyond the research setting were generated. The most important findings are discussed below.

The first research question was about assessing whether SbS is considered acceptable, relevant, and beneficial among the Lebanese and Syrians in Lebanon. It was evident throughout this qualitative assessment that this was true for the Lebanese and the displaced Syrians with depression. With the deteriorating mental health of the population at large, due to the multiple humanitarian and economic crises and the worsening living conditions in Lebanon, the need for accessible, feasible, evidence-based, and free-of-charge mental health services is evident. Refugees in particular are highly prone to distress and common mental health disorders due to the displacement and stressors they endure (30, 31). Findings suggest that SbS might address some of the core barriers to seeking adequate mental health care among the displaced and host populations. It created access for people who would normally not have been able to get treatment due to stigma, lack of services, increased poverty levels, social distancing regulations, and the fuel shortage crises (23, 24, 32). As discussed by key informants, SbS was expected to tend to the needs of a big portion of the population such as young adults, the tech-savvy, the literate, and those with access to smartphones. It was also expected to be more acceptable and trusted by those who are exposed to mental health awareness, hence the need to increase mental health literacy among those residing in Lebanon to promote the acceptability and uptake of digital interventions (2, 11). Nonetheless, there is no “one solution fits all,” and digital interventions will not be accessible to everyone. It is thus recommended to integrate SbS in a system with a mix of evidence-based services in face-to-face format or other outreach methods and material, accessible to those who don't use the digital platforms for treatment.

This paper also sheds light on the exercises and features that were mostly accepted, perceived as beneficial, and feasible to apply by the participants. Straightforward techniques seemed to be more accepted than more complex and multi-step exercises among Lebanese and Syrian participants of the SbS intervention in Lebanon. Participants found those exercises easy to grasp and apply and were able to see tangible and direct benefits after practicing them. Those exercises included small “self-care activities,” the “breathing exercise,” a “gratitude list,” and “positive self-talk.”

In contrast, managing the “more challenging daily activities” that included several smaller steps and layers, the calendar scheduling, and the social activities that involved effort and reaching out to others, were perceived as more complicated to apply. These findings are relevant to consider when designing digital self-help treatments as they could impact the usability of the intervention, and the motivation to complete it (2, 11).

Another important consideration regarding the usability and uptake of digital interventions is that the real impact might be difficult to track or evaluate. Many users mentioned that they didn't input their activities online as some found the calendar and the interactive part of the platform complex. This was validated by the usage metrics generated in previous research phases, where most respondents didn't input the challenging daily activities into the online interactive platform (22). Yet, throughout this evaluation, we learned that some used these techniques offline on their personal agendas or offline calendars or implemented them in their daily life after completing the program. This finding suggests that there might be a greater impact of digital intervention on users that is not properly depicted in quantitative studies or in the dropout rates reported as it cannot be documented in the application. It further suggests the importance of understanding existing user behavior and ensuring applications are as user-friendly and easy to use as possible.

The second research question focused on assessing the uptake of SbS in Lebanon and uncovering the main enabling factors and barriers for retaining participants in the research study and preventing further dropout from the intervention. High dropout is not a unique problem to SbS; research highlights the low uptake of digital interventions and smartphone apps despite the wide use of smartphones and the internet globally and in LMICs (1). Studies have shown that dropout rates can reach up to 52% during sign-up to digital interventions, 78% during the treatment phase, and 18% during follow-ups (12). SbS was not an exception, with high dropout rates, of 46.2% for Syrians and 65.1% for Lebanese, witnessed during the RCTs (23, 24). This paper described the set of challenges related to the SbS digital intervention that hindered the retention of users. In congruence with other implementation studies on digital mental health interventions, barriers to adherence and uptake were categorized as intervention-related, research-related, and user-related (2, 11). At the intervention level, many problems in the usability and bugginess of the application were revealed in the SbS intervention. Although the appealing design, features, colors, and easy navigation in the application were highly praised by users, the poor usability and bugginess of the application was a major barrier to adherence to the experience of the users and that of the e-helpers. Challenges included forgetting the passwords, lack of available space to download the app, slow internet connectivity, bugginess, and technical problems encountered, among others. The poor usability of the digital interventions is highlighted in the literature as a common variable affecting dropout, hence the importance to design user-centric interventions that provide a smooth and seamless journey for the users (2, 12, 33). Other barriers encountered were research-related and included demotivation to fill the long and burdening assessments and the time taken to enter data and sign-up for the intervention. This was also consistent with the literature on digital mental health research and dropout associated with the burden of assessments (2, 11, 12). Although assessments could be shortened during the scale-up, they could still pose a problem in digital research trials. The latter could be long, tedious, and repetitive, which could cause people to get impatient and stop answering them. It's thus worth exploring the modality of administering them and pacing them into smaller chunks or adding motivational messages for the users to complete them.

Additionally, user-related barriers were debunked during this evaluation, such as lack of inner motivation or perceived benefit, competing priorities, and busy lifestyles, among others. The lack of motivation is a key symptom of depression as highlighted in the literature and thus more motivation and engagement are required, as well as ensuring that the intervention is brief and efficient (2). This underlines the important role of the e-helpers' guidance and motivation and the need to preserve it for scale-up. On the other hand, perceived benefit of the intervention and high digital literacy were highlighted as user-related factors that promote adherence in a recent systematic review (33). This was comparable to the results depicted in the Step-by-Step evaluation.

In terms of promoting factors related to the intervention, our evaluation results showed that participants valued the cultural relevance of the content and its relatability. This could imply that the extensive user-testing and cultural adaptation previously conducted led to a user-friendly design and relevant content (story, illustrations, local idioms, examples, and exercises) (19). These were crucial to address the poor usability and the lack of user-centric approaches that were identified as barriers to adherence in different studies (2). Other promoting factors for adherence were related to the delivery model and follow-up approach in SbS. This entailed engaging push notifications and automated reminders, as well as active follow-up by the e-helpers. These engagement factors of the digital intervention and the team are highlighted in the literature as a best practice to engage users and retain them (11, 33). Participants in the intervention group praised the important role of the e-helpers in motivating them, supporting them, and getting them to commit to the program through the “oral contract” in the introductory call. Positive feedback and gratitude messages are recognized as promoting factors for adherence to digital interventions (11). From a research perspective, the phone credit compensations given for participation to cover the internet data costs were mentioned as an effective incentive among Syrians. They highlighted the importance of receiving smaller installments of compensation at several points upon completion of the pre, post, and follow-up assessments, instead of getting one lump sum upon completion at 5 months as was done in previous phases. The paced installments served as reminders and incentives for them to continue and helped them better manage their data consumption in using the application. Due to the worsening financial situation and the harsh conditions endured, taking part in the research might be a way to receive financial compensation. This finding is worth exploring as a best practice during research projects. Yet, it is noteworthy that in the scale-up model, there won't be any financial compensation. Hence the need to manage expectations, ensure internet access, and stress the other benefits of the program such as the free treatment concept.

The third research question investigated the challenges and recommended modalities for sustaining SbS in Lebanon. Despite the acknowledgment of the need, relevance, and benefits of SbS among the Lebanese and the displaced populations in Lebanon, many concerns and challenges were foreseen by different stakeholders in maintaining the intervention in the long run. The barriers identified both at the project and contextual levels were retaining staff amidst the increasing brain drain, securing sustainable funds to cover the running costs of the intervention amidst competing international priorities, and accessing smartphones and the Internet in light of a deteriorating infrastructure and a severe economic crisis in Lebanon (34). The current humanitarian crisis presents different types of requirements for the sustainability of digital mental health interventions with minimal guidance. For instance, Lebanon witnessed a doubled increase in unemployment rates between 2019 and 2022 and an increase in brain drain (35, 36). Consequently, the volunteering model of the e-helpers, previously planned, might not be practical during an economic crisis where young adults and fresh graduates would tend to look for paid internships or job opportunities instead of volunteering for free. Different scenarios such as employment models or internship opportunities would need further exploration for the scale-up phase. Similarly, securing international funds and exploring local modalities for sustaining SbS as a governmental free-of-charge service is crucial for the maintenance of the service. The recommendations in Supplementary material 5 hold crucial suggestions for the maintenance, sustainability, and integration of SbS in the mental health care system in Lebanon amidst this volatile phase.

### Study limitations

The main limitation of this study was the difficulty to reach people who dropped out to garner more feedback and recommendations. These participants were unresponsive to the contacts of the study team. Another limitation was the possible bias generated during the interviews since most of the interviewers were affiliated with SbS directly or indirectly. Finally, the discrepancy of interview methods for SbS staff (face-to-face) and SbS participants and external stakeholders (phone) paused a limitation to the methodology.

#### What this study adds

The potential of digital mental health interventions is under-researched in LMICs among vulnerable groups and populations affected by adversities. This qualitative study helps bridge this gap by providing an insight into the uptake, acceptability, and feasibility of a digital mental health intervention in a LMIC setting, among vulnerable and displaced populations. By taking a closer look into the users, staff, and stakeholders' perspectives, this research uncovered significant barriers and enabling factors for the adherence and uptake of digital mental health interventions in such settings. Findings included crucial considerations and best practices for the implementation and scale-up of Step-by-Step in Lebanon. This paper's findings complement the effectiveness and cost-effectiveness evaluation of SbS and provide practical feedback and recommendations to ensure a proper uptake and successful implementation of a user-centered service. Researchers and project managers can benefit from the findings in this paper to make use of the design, implementation, and scale-up of digital interventions under challenging settings.

## Conclusion

Digital mental health interventions have great potential in improving the quality and accessibility of mental health services in low-and-middle-income countries, considering their practical use, easy dissemination, and low cost. Given their proven effectiveness, high hopes exist for these interventions to bridge the mental health treatment gap for people in need. Nevertheless, their uptake remains low, and adherence to online treatment is widely documented as a universal challenge. Research about the key barriers and enabling factors for their feasibility and acceptability among vulnerable groups, displaced populations, and those affected by adversities in low-resource settings is still limited. This qualitative evaluation analyzed the acceptability and feasibility of a digital mental health intervention for depression among Lebanese and Syrian displaced populations exposed to adversities in Lebanon. It also investigated the reasons for the high dropout encountered and shared the promoting factors for adherence. Recommendations and best practices were generated and aimed to guide the scale-up of SbS in Lebanon. The study's findings and recommendations can help explore how digital mental health interventions could be leveraged to improve access to care among people with mental disorders in low-resource settings. Findings are relevant to researchers, implementers, and policymakers to ensure a successful and sustained roll-out of such interventions upon scale-up.

## Data availability statement

The datasets presented in this article are not readily available because, they contain unique information or a combination of answers that might reveal the identity of the interviewees and could risk breaching their confidentiality. The consequences of such breaches are rated as severe for the users specifically. Requests to access the datasets should be directed to JAR, jinane.abiramia@gmail.com.

## Ethics statement

The studies involving humans were approved by WHO Research Ethics Review Committee and St Joseph's University Ethics Committee in Beirut (CEHDF 862). The studies were conducted in accordance with the local legislation and institutional requirements. Written informed consent for participation was not required from the participants or the participants' legal guardians/next of kin because vulnerable groups interviewed included adults living in Lebanon and suffering from depression, as well as Syrian refugees. All participants displayed autonomy and capacity to provide written online consent through the app platform upon signing up and through the messaging platform before conducting the interview, and once again orally at the beginning of the phone interview (audio-recorded).

## Author contributions

JAR: Conceptualization, Data curation, Formal analysis, Investigation, Methodology, Project administration, Writing – original draft, Writing—review & editing. RAH: Data curation, Formal analysis, Investigation, Methodology, Validation, Writing—review & editing. PN: Data curation, Investigation, Methodology, Project administration, Writing—review & editing. PC: Conceptualization, Methodology, Supervision, Writing—review & editing. KC: Conceptualization, Funding acquisition, Methodology, Project administration, Supervision, Writing—review & editing. EvH: Conceptualization, Funding acquisition, Methodology, Project administration, Supervision, Writing—review & editing. EH: Conceptualization, Project administration, Writing—review & editing. EZ: Conceptualization, Funding acquisition, Project administration, Supervision, Writing—review & editing. MS: Conceptualization, Methodology, Supervision, Writing—review & editing. REC: Conceptualization, Funding acquisition, Methodology, Project administration, Supervision, Writing—review & editing.

## Supplementary material

Supplementary material 1 Informed consent form - study participants.

Supplementary material 2 Interview guide with external stakeholders.

Supplementary material 3 Interview guide with staff.

Supplementary material 4 Interview guide with study participants.

Supplementary material 5 Table 2. recommendations.
