# Supplementary material for: Feasibility and uptake of a digital mental health intervention for depression among Lebanese and Syrian displaced people in Lebanon: a qualitative study
Source: Front Public Health. 2024 Jan 22;11:1293187. doi: 10.3389/fpubh.2023.1293187 (PMC10840138; doi:10.3389/fpubh.2023.1293187)
Supplement: Supplementary material 1 — Informed consent form - study participants. [file Data_Sheet_1.pdf]

CONSENT FORM Interview for SBS RCT Evaluation

Informed Consent Form- Study participants (online)

This Informed Consent Form is for participants in Step-by-Step who we are inviting to participate in qualitative research on local adaptation of an internet-based health programme. The title of our research project is Step-by-Step.

Site principal investigator: Dr Rabih El Chammay, Ministry of Public Health, Lebanon. Sponsor: World Health Organization

This Informed Consent Form has two parts:

- **Information Sheet (to share information about the research with you)**
- **Certificate of Consent (for signatures if you agree to take part)**

You will be given a copy of the full Informed Consent Form

PART I: Information Sheet

Introduction

We are a group of researchers from the Ministry of Public Health. This research project is being done with the World Health Organization, and supported by a charitable foundation in Switzerland. We have developed a new, internet-based support programme for people experiencing difficult emotions. This programme is called "Step by Step".

As a participant in this research project, we invite you to take part in the evaluation interview to inform the future phases of the project.

Purpose of the research

As you know, we have designed a programme to help people living in this area who are dealing with difficult emotions. Difficult emotions, such as feelings of sadness or stress are very common, especially when people are struggling with problems in their lives.

Now that you've been involved in our programme, we believe that you can help us by telling us your thoughts about the programme, both good and bad. We would like you to be honest about the programme, in order to help us to improve it.

Type of Research Intervention

This research will involve your participation in one interview that will take about 45 minutes.

Participant Selection

You are being invited to take part in this research because you have had experience with the programme in some way and your experience can help us to make the programme better.

- ***Question to check understanding:*** *Do you know why we are asking you to take part in this research?*

Voluntary Participation and Right to Withdraw

CONSENT FORM Interview for SBS RCT Evaluation

Your participation in this research is voluntary: That means that it is your choice whether or not to be in this research project. No one is forcing you. You may change your mind at any time. You can stop the interview at any time, and you can also ask that your answers are not included in the research. Deciding not to participate, or stopping the interview, will not have any negative impact on you or your work.

At the end of the interview, if there is something that you said that you wish to not be included in the research, you can tell us and we will leave it out. If there is something you want to change about what you said, you can also tell us that, and we will change it in our notes.

> ***Questions to check understanding:*** *Do you know that you do not have to take part in this research project, if you do not wish to?*

Procedures
If you agree, we will ask you some questions about your experiences with the programme.

We will ask you to have an interview with an interviewer from the Ministry of Public Health Lebanon. The interviewer will conduct a phone interview with you on a date and time that you both agree on. If you do not wish to answer any of the questions during the interview, you may say so and the interviewer will move on to the next question. The interviewer will not be upset if you have any negative comments about the programme.

No one else but the interviewer will be on the phone, unless you would like someone else to be there. The interviewer will use a recorder to record the interview, so that we can listen again later to what you told us. We will keep this recording safe. The first interview will last for about 45 minutes. If we need some more time to ask all of the questions, or if you have more information to give us, the interviewer will arrange a time to meet again for a second interview.

Questions to check understanding : *If you decide to take part in the study, do you understand what would be involved? Do you know that you may not answer the questions that you do not wish to respond to?*

Risks
If you find any of the questions too personal, uncomfortable or difficult to answer you do not have to answer them.

You do not have to give us any reason for not answering any question, or for not completing the interview. If you become very upset from talking about your experience of the programme, you will be able to speak to a member of staff who will support you.

Benefits
There may not be any benefits to you from completing this interview, but your participation is likely to help us to develop a more useful programme to help local people in managing emotional problems.

Reimbursements
You will not receive any compensation for participating in the interview research.

CONSENT FORM Interview for SBS RCT Evaluation

- ***<u>Questions to check understanding:</u>*** *Can you tell me if you have understood correctly the benefits that you will have if you take part in the interview? Do you have any other questions?*

Confidentiality
All researchers working on this project will ensure privacy and confidentiality for all information and documents from the research.

All information collected about you will be kept strictly confidential. What we record on the recorder will be listened to and written down, and we will not write down any names. Once it is written down, we will erase this recording.

Any information about you will have a number on it instead of your name, and a list linking your name to your number will be kept safe. Data will be stored on a computer and only the research team will have access to it. Any results that we report will not reveal the identity of any participants.
The data will be destroyed after five years.

- ***<u>Questions to check understanding:</u>*** *Do you understand how we will make sure that any information that we as collect about you will remain confidential? Do you have any questions about this?*

Sharing the Results
The knowledge that we gain during this research will be shared in a general only. We will publish what we have learnt so that other interested people may learn from this research, but we will never include information which would identify you as having taken part. The information we gain will be used to further develop the programme.

Who to Contact
If you have questions now you can ask me. We will also give you the name and phone number of a study team member to contact if you have questions later. This person is: Dr Rabih El Chammay, Ministry of Public Health, Phone no: 01611672, ext: 125.

This proposal has been reviewed and approved by the Institution Review Board of the Saint Joseph University, Lebanon. This is a group of people whose task it is to make sure that research participants are protected from harm. If you wish to find about more about the committee, contact Dr Rabih El Chammay, Ministry of Public Health, on 01611672, ext: 125. This proposal has also been reviewed by the ethics review committee of the World Health Organization (WHO), which is supporting the study.

<u>Questions to check understanding:</u> *Do you know how we will share the results of the study?*

<u>Question:</u> *You can ask me now, or call or email us later to ask any questions about any part of the research study, if you wish to. Do you have any questions?*

<u>Part II: Certificate of Consent</u>
I have been invited to participate in research about emotional problems in our community.

CONSENT FORM Interview for SBS RCT Evaluation

I have read and understood the above information, or it has been read to me. I have had the opportunity to ask questions and any questions I have been asked have been answered to my satisfaction. I consent voluntarily to be a participant in this study.

Name of Participant____

Signature of Participant ____

Date ___________________________

Day/month/year

Informed Consent- Project Staff (face to face)

This Informed Consent Form is for project staff who we are inviting to participate in qualitative research on local adaptation of an internet-based health programme. The title of our research project is Step-by-Step.

Site principal investigator: Dr Rabih El Chammay, Ministry of Public Health, Lebanon. Sponsor: World Health Organization

This Informed Consent Form has two parts:

- **Information Sheet (to share information about the research with you)**
- **Certificate of Consent (for signatures if you agree to take part)**

You will be given a copy of the full Informed Consent Form

PART I: Information Sheet

Introduction

We are a group of researchers from the Ministry of Public Health. This research project is being done with the World Health Organization, and supported by a charitable foundation in Switzerland. We have developed a new, internet-based support programme for people experiencing difficult emotions. This programme is called "Step by Step".

As a key stakeholder in this research project, we invite you to take part in the evaluation interview to inform the future phases of the project.

Purpose of the research

As you know, we have designed a programme to help people living in this area who are dealing with difficult emotions. Difficult emotions, such as feelings of sadness or stress are very common, especially when people are struggling with problems in their lives.

Now that you've been involved in advising or disseminating our programme, we believe that you can help us by telling us your thoughts about the programme, both good and bad. We would like you to be honest about the programme, in order to help us to improve it.

CONSENT FORM Interview for SBS RCT Evaluation

Type of Research Intervention
This research will involve your participation in one interview that will take about 45 minutes.

Participant Selection
You are being invited to take part in this research because you have had experience with the programme in some way and your experience can help us to make the programme better.

- ***Question to check understanding:*** *Do you know why we are asking you to take part in this research?*

Voluntary Participation and Right to Withdraw
Your participation in this research is voluntary: That means that it is your choice whether or not to be in this research project. No one is forcing you. You may change your mind at any time. You can stop the interview at any time, and you can also ask that your answers are not included in the research. Deciding not to participate, or stopping the interview, will not have any negative impact on you or your work.

At the end of the interview, if there is something that you said that you wish to not be included in the research, you can tell us and we will leave it out. If there is something you want to change about what you said, you can also tell us that, and we will change it in our notes.

Questions to check understanding: *Do you know that you do not have to take part in this research project, if you do not wish to?*

Procedures
If you agree, we will ask you some questions about your experiences with the programme.

We will ask you to have an interview with an interviewer from the Ministry of Public Health Lebanon. The interviewer will conduct a phone interview with you on a date and time that you both agree on. If you do not wish to answer any of the questions during the interview, you may say so and the interviewer will move on to the next question. The interviewer will not be upset if you have any negative comments about the programme.

No one else but the interviewer will be in the room, unless you would like someone else to be there. The interviewer will use a recorder to record the interview, so that we can listen again later to what you told us. We will keep this recording safe. The first interview will last for about 45 minutes. If we need some more time to ask all of the questions, or if you have more information to give us, the interviewer will arrange a time and place to meet again for a second interview.

Questions to check understanding : *If you decide to take part in the study, do you understand what would be involved? Do you know that you may not answer the questions that you do not wish to respond to?*

Risks
If you find any of the questions too personal, uncomfortable or difficult to answer you do not have to answer them.

You do not have to give us any reason for not answering any question, or for not completing

the interview. If you become very upset from talking about your experience of the programme, you will be able to speak to a member of staff who will support you.

Benefits

There may not be any benefits to you from completing this interview, but your participation is likely to help us to develop a more useful programme to help local people in managing emotional problems.

Reimbursements

You will not receive any compensation for participating in the interview research.
However, any costs for you to come to the interview, and to bring another person if you would like someone to be there with you, will be paid for by us.

- ***<u>Questions to check understanding:</u>*** *Can you tell me if you have understood correctly the benefits that you will have if you take part in the interview? Do you know if the study will pay for travel costs? For how many people? Do you have any other questions?*

Confidentiality

All researchers working on this project will ensure privacy and confidentiality for all information and documents from the research.

All information collected about you will be kept strictly confidential. What we record on the recorder will be listened to and written down, and we will not write down any names. Once it is written down, we will erase this recording.

Any information about you will have a number on it instead of your name, and a list linking your name to your number will be kept safe. Data will be stored on a computer and only the research team will have access to it. Any results that we report will not reveal the identity of any participants.
The data will be destroyed after five years.

- ***<u>Questions to check understanding:</u>*** *Do you understand how we will make sure that any information that we as collect about you will remain confidential? Do you have any questions about this?*

Sharing the Results

The knowledge that we gain during this research will be shared in a general only. We will publish what we have learnt so that other interested people may learn from this research, but we will never include information which would identify you as having taken part. The information we gain will be used to further develop the programme.

Who to Contact

If you have questions now you can ask me. We will also give you the name and phone number of a study team member to contact if you have questions later. This person is: Dr Rabih El Chammay, Ministry of Public Health, Phone no: 01611672, ext: 125.

This proposal has been reviewed and approved by the Institution Review Board of the Saint

CONSENT FORM Interview for SBS RCT Evaluation

Joseph University, Lebanon. This is a group of people whose task it is to make sure that research participants are protected from harm. If you wish to find about more about the committee, contact Dr Rabih El Chammay, Ministry of Public Health, on 01611672, ext: 125. This proposal has also been reviewed by the ethics review committee of the World Health Organization (WHO), which is supporting the study.

> ***Questions to check understanding:*** *Do you know how we will share the results of the study?*

Question: *You can ask me now, or call or email us later to ask any questions about any part of the research study, if you wish to. Do you have any questions?*

Part II: Certificate of Consent

I have been invited to participate in research about emotional problems in our community.
I have read and understood the above information, or it has been read to me. I have had the opportunity to ask questions and any questions I have been asked have been answered to my satisfaction. I consent voluntarily to be a participant in this study.

Print Name of Participant____

Signature of Participant ____

Date ____________________________
Day/month/year

Informed Consent- External Stakeholders (online)

This Informed Consent Form is for stakeholders who we are inviting to participate in qualitative research on local adaptation of an internet-based health programme. The title of our research project is Step-by-Step.

Site principal investigator: Dr Rabih El Chammay, Ministry of Public Health, Lebanon. Sponsor: World Health Organization

This Informed Consent Form has two parts:

- **Information Sheet (to share information about the research with you)**
- **Certificate of Consent (for signatures if you agree to take part)**

You will be given a copy of the full Informed Consent Form

PART I: Information Sheet

Introduction

We are a group of researchers from the Ministry of Public Health. This research project is being done with the World Health Organization, and supported by a charitable foundation in Switzerland. We have developed a new, internet-based support programme for people

experiencing difficult emotions. This programme is called "Step by Step".

As a key stakeholder in this research project, we invite you to take part in the evaluation interview to inform the future phases of the project.

Purpose of the research

As you know, we have designed a programme to help people living in this area who are dealing with difficult emotions. Difficult emotions, such as feelings of sadness or stress are very common, especially when people are struggling with problems in their lives.

Now that you've been involved in advising or disseminating our programme, we believe that you can help us by telling us your thoughts about the programme, both good and bad. We would like you to be honest about the programme, in order to help us to improve it.

Type of Research Intervention
This research will involve your participation in one interview that will take about 45 minutes.

Participant Selection
You are being invited to take part in this research because you have had experience with the programme in some way and your experience can help us to make the programme better.

- ***Question to check understanding:*** *Do you know why we are asking you to take part in this research?*

Voluntary Participation and Right to Withdraw
Your participation in this research is voluntary: That means that it is your choice whether or not to be in this research project. No one is forcing you. You may change your mind at any time. You can stop the interview at any time, and you can also ask that your answers are not included in the research. Deciding not to participate, or stopping the interview, will not have any negative impact on you or your work.

At the end of the interview, if there is something that you said that you wish to not be included in the research, you can tell us and we will leave it out. If there is something you want to change about what you said, you can also tell us that, and we will change it in our notes.

Questions to check understanding: *Do you know that you do not have to take part in this research project, if you do not wish to?*

Procedures
If you agree, we will ask you some questions about your experiences with the programme.

We will ask you to have an interview with an interviewer from the Ministry of Public Health Lebanon. The interviewer will conduct a phone interview with you on a date and time that you both agree on. If you do not wish to answer any of the questions during the interview, you may say so and the interviewer will move on to the next question. The interviewer will not be upset if you have any negative comments about the programme.

CONSENT FORM Interview for SBS RCT Evaluation

No one else but the interviewer will be on the phone, unless you would like someone else to join. The interviewer will use a recorder to record the interview, so that we can listen again later to what you told us. We will keep this recording safe. The first interview will last for about 45 minutes. If we need some more time to ask all of the questions, or if you have more information to give us, the interviewer will arrange a time to meet again for a second interview.

Questions to check understanding : *If you decide to take part in the study, do you understand what would be involved? Do you know that you may not answer the questions that you do not wish to respond to?*

Risks

If you find any of the questions too personal, uncomfortable or difficult to answer you do not have to answer them.

You do not have to give us any reason for not answering any question, or for not completing the interview. If you become very upset from talking about your experience of the programme, you will be able to speak to a member of staff who will support you.

Benefits

There may not be any benefits to you from completing this interview, but your participation is likely to help us to develop a more useful programme to help local people in managing emotional problems.

Reimbursements

You will not receive any compensation for participating in the interview research.

- ***Questions to check understanding:*** *Can you tell me if you have understood correctly the benefits that you will have if you take part in the interview? Do you have any other questions?*

Confidentiality

All researchers working on this project will ensure privacy and confidentiality for all information and documents from the research.

All information collected about you will be kept strictly confidential. What we record on the recorder will be listened to and written down, and we will not write down any names. Once it is written down, we will erase this recording.

Any information about you will have a number on it instead of your name, and a list linking your name to your number will be kept safe. Data will be stored on a computer and only the research team will have access to it. Any results that we report will not reveal the identity of any participants.

The data will be destroyed after five years.

- ***Questions to check understanding:*** *Do you understand how we will make sure that any information that we as collect about you will remain confidential? Do you have any questions about this?*

CONSENT FORM Interview for SBS RCT Evaluation

Sharing the Results

The knowledge that we gain during this research will be shared in a general only. We will publish what we have learnt so that other interested people may learn from this research, but we will never include information which would identify you as having taken part. The information we gain will be used to further develop the programme.

Who to Contact

If you have questions now you can ask me. We will also give you the name and phone number of a study team member to contact if you have questions later. This person is: Dr Rabih El Chammay, Ministry of Public Health, Phone no: 01611672, ext: 125.

This proposal has been reviewed and approved by the Institution Review Board of the Saint Joseph University, Lebanon. This is a group of people whose task it is to make sure that research participants are protected from harm. If you wish to find about more about the committee, contact Dr Rabih El Chammay, Ministry of Public Health, on 01611672, ext: 125. This proposal has also been reviewed by the ethics review committee of the World Health Organization (WHO), which is supporting the study.

Questions to check understanding: *Do you know how we will share the results of the study?*

Question: *You can ask me now, or call or email us later to ask any questions about any part of the research study, if you wish to. Do you have any questions?*

Part II: Certificate of Consent

I have been invited to participate in research about emotional problems in our community.
I have read and understood the above information, or it has been read to me. I have had the opportunity to ask questions and any questions I have been asked have been answered to my satisfaction. I consent voluntarily to be a participant in this study.

Print Name of Participant____

Signature of Participant ____

Date ____________________________

Day/month/year
