# Supplementary material for: Feasibility and uptake of a digital mental health intervention for depression among Lebanese and Syrian displaced people in Lebanon: a qualitative study
Source: Front Public Health. 2024 Jan 22;11:1293187. doi: 10.3389/fpubh.2023.1293187 (PMC10840138; doi:10.3389/fpubh.2023.1293187)
Supplement: Supplementary material 2 — Interview guide with external stakeholders. [file Data_Sheet_2.pdf]

Interview guide with external stakeholders- (online)

Increasing Access to Mental Health Care for People in Lebanon Living in Adversity:

"Step-by-Step" intervention

Add intro about SBS:

The "Step-by-Step" intervention is an evidence-based, innovative approach to treatment for depression using a five-session internet-delivered self-help intervention for adults experiencing common mental health issues. The World Health Organization (WHO) and the Ministry of Public Health (MoPH) tested its feasibility and effectiveness through a Randomized Controlled Trial (RCT) in Lebanon and now it is available as a service. People interested in this program can access Step-by-Step through their own device (app or web-browser). "Step by Step" users will receive technical and motivational support each week from e-helpers, who are non-specialists trained to provide basic support for the users. The National Mental Health Programme (NMHP) at MoPH is planning to scale it up into a national service, if the results of the RCT are positive. We would like to get your feedback and insight on the feasibility of such e-mental health intervention in Lebanon.

<u>Qualitative Evaluation Interview Guide</u>

1. **General feedback**

    a. Based on your understanding of SbS (self-help app with guidance from a trained helper), are you satisfied / supportive of the model and approach taken? Are there any changes you would suggest for the app, its content or the overall system? what parts do you think are the most or least helpful?

    b. What were the feedback/impressions you heard about SBS? From whom?

2. **Feasibility, acceptability and relevance**

    a. How feasible do you think will it be to implement SBS as a service (e.g. outside of research)_in Lebanon?

    b. To what extent do you think this service will be acceptable among the Lebanese, and among different populations residing in Lebanon (Syrians, Palestinians)?

    c. To what extent do you think SBS would be helpful or relevant to the people residing in Lebanon? Prob: if they answer yes, can you explain why you think it will be helpful or useful?

    d. Are there any population groups it would be more or less feasible, acceptable or relevant to? (age, nationality, literacy, etc…)

    e. Are there any changes you would suggest that might increase its feasibility, acceptability or relevance when scaled up?

3. **Implementation and project placement in Lebanon**

a. NMHP are planning to run SbS in a similar way to the national hotline, with volunteering e-helpers, etc... Do you have any suggestions on how we could make this model successful? in addition, could you see any way we can integrate it into other services. What would they be?

b. Who are the stakeholders that we would need to involve in order to run SbS or to ensure SbS is well disseminated amongst networks? (public, private sector, NGOs, syndicates, etc…). What would the role of NMHP be after the implementation piloting?

4. Foreseen challenges, and recommendations

a. Two challenges we faced during the research were recruitment (ensuring people knew about the service and its availability), ensuring people did not drop out. do you have any suggestions on how we might address these challenges?

b. What other challenges do you foresee in the scale up of SBS in Lebanon? How do you think can we tackle/avoid those challenges? (cost, e-helper volunteering model, etc..)

c. What recommendations do you suggest for a better implementation of the service and higher effectiveness and reach?

d. Are you involved or did you learn about any public health application that is being prepared or delivered for the community? (Not necessarily mental health) or remote support services? If yes, what are some lessons learned?
