# Supplementary material for: Feasibility and uptake of a digital mental health intervention for depression among Lebanese and Syrian displaced people in Lebanon: a qualitative study
Source: Front Public Health. 2024 Jan 22;11:1293187. doi: 10.3389/fpubh.2023.1293187 (PMC10840138; doi:10.3389/fpubh.2023.1293187)
Supplement: Supplementary material 3 — Interview guide with staff. [file Data_Sheet_3.pdf]

Interview guide with project staff- (face to face)

Increasing Access to Mental Health Care for People in Lebanon Living in Adversity: "Step-by-Step" intervention

Greet person. Introduce self, including what organization you are working for. Explain the study, following written informed consent process.

Possible additional explanation of semi-structured interview process:

You have recently participated as an e-helper in the Step-by-Step study. We would like to ask you some questions about your experience with the study and the platform, to help us to think about how Step-by-Step and the procedures could be improved for delivery in the future.

There are no right or wrong answers to the questions we are going to ask. The interview will be recorded without any identifier to make sure that answers are transcribed accurately; all answers will be reported anonymously to ensure confidentiality. We will be speaking to a number of people, asking everyone the same questions. If you feel unable to answer a question please say and we will move on to the next one.

In note book document date and site of interview, age and gender of interviewee, who they are (i.e. intervention participant, helper, policy maker), and initials of interviewers.

Begin semi-structured interview:

Record responses and take down pertinent responses in the notebook.

Interview process:

- <u>Overall impressions:</u>
    - 1- Can you describe your experience with Step-by-Step and the study in general. Explore positive / negative views through probes.
        - How was your experience in being part of a research study?
        - How was your experience in being part of electronic self-help program?
        - Have you encountered any technical issues and if yes, get a short description of the issue using the probes:
          1) What did you do?
          2) What did you expect would happen?
          3) What did actually happen?
        - How much do you believe that an electronic guided self-help service can be useful/accepted in Lebanon?
    - 2- Please think back to your experience of using the website/app and tell us about your experience using the app, including features and design. (how was it for you to navigate through the sessions, exercises, did you like the colors, etc..)
        - What are the features/exercises you liked the most?
        - What are the features/exercises you didn't like and why?
        - What alternative features or options do you recommend to have? To help improve your experience?
- <u>Experience of clients:</u>
    - 3- Can you describe your rapport with clients you have supported?
        - Explore positive / negative views through probes. (If they are not forthcoming to open questions, can ask about the following: follow up with users, relationship, most encountered challenges, difficulties, positive changes)

Interview guide with project staff- (face to face)

- 4- Did you encounter any difficulties with families of clients you have supported or suspect any risk issues arising from communications with your clients?
  - Explore positive / negative views through probes
  - 5- Do you think users will be able to use Step-by-Step without the e-helpers support at some point?
  - 6- Are there any changes required to improve the users' experience in this program? (adding more calls per week, longer calls? Chatting versus messaging?)

- 7- Are there any changes or suggestions you have for the helper model?
  - Explore positive / negative views through probes. – any changes they would advise or suggest? Anything that was liked or not liked?

- <u>Intervention adherence:</u>
  - 8- Based upon your experience, what would help people stay motivated to engage with Step-by-Step and practice or use some of the techniques.
  - 9- To what extent do you feel your clients implemented the skills they learned in the programme? Explore barriers and facilitators to skills development
    - What are the features/activities they liked the most?
    - What are the least used features? Why?
    - (did they download the audio option? did they read the story? Mood tracker, calendar, gratitude, small versus complex activities, audio exercises?)
    - Was there any preferred session among your users? Or a session they didn't like? Why?
    - What are alternative options or features that users suggested?
    - How much were the technical problems encountered hindering their adherence?
    - What were some common reasons for dropout or unresponsiveness?
    - What helped in improving the adherence? (compensations divided into 3, etc..?)
  - 10- How could we encourage users to use SbS as intended e.g. reading the story or completing the exercises?

  - 11- Is there anything else we could do to help users stay motivated?
    - Explore further if they are not clear.

- <u>Local management and supervision:</u>
  - 12- What is your feedback on your shift and time/workload management during the shift?
    - Caseload – what do you think is a manageable number per day / shift?
    - Is the 15 mins reasonable? Did it take you some time for that to feel ok, if so, how can we support with that?
    - Is there anything that was not necessary or that needed to be amended? (case notes/etc..)
  - 13- What is your overall impression on the supervision model (weekly meetings/face to face) how do you describe its effectiveness in solving problems encountered? (were you able to discuss and solve challenges in your team meetings?)

Interview guide with project staff- (face to face)

  - What could be improved?
  - 14- what would you add or change about the helper manual and other materials (SOPs, etc..)? is there anything that you wish was there? More guides, tips, processes, templates?
  - 15- Based on your experience, how was the orientation phase including training and support provided during the initiation phase? Was the training length and structure acceptable? (3 days theory about research and intervention and 3 half days role plays). Do you recommend any changes? is it sufficient and clear to start the implementation phase directly or is there a need for further training? (Would you suggest any changes?)
  - 16- What do you need to get comfortable in your role? Looking back, anything you would have liked to know at the start?

1. Review any written records with the interviewee still present. If anything is not clear, ask for clarification and correct written notes as necessary.

2. Ask the interviewee if they have anything to add. Any additional information is added to the interview notes as required.

3. Thank person and leave.

Interview Guide: Clinical/admin supervisor

Greet person. Introduce self, including what organization you are working for. Explain the study, following written informed consent process.
Possible additional explanation of semi-structured interview process:
You have recently participated as clinical supervisor in the Step-by-Step study. We would like to ask you some questions about your experience with the programme and the study, to help us to think about how Step-by-Step and the procedures could be improved for delivery in the future.
There are no right or wrong answers to the questions we are going to ask. The interview will be recorded to make sure that answers are transcribed accurately; all answers will be reported anonymously to ensure confidentiality. We will be speaking to a number of people, asking everyone the same questions. If you feel unable to answer a question please say and we will move on to the next one.

In note book document date and site of interview, age and gender of interviewee, position and initials of interviewer.

Begin semi-structured interview:
Record responses and write pertinent responses in the notebook.
Interview process:

- Overall impressions:
  - 1- Can you describe your experience with Step-by-Step and the study in general?
    - Explore positive / negative views through probes.
    - How was your experience in being part of a research study?
    - How was your experience in being part of electronic self-help program?
    - How much do you believe that an electronic self-help service can be useful/accepted in Lebanon?

Interview guide with project staff- (face to face)

- 2- Please think back to your experience of using the website/app and tell us about your experience using the app, including features and design. (how was it for you to navigate through the sessions, exercises, did you like the colors, etc...)
    - What are the features/exercises you liked the most?
    - What are the features/exercises you didn't like and why?
    - What alternative features or options do you recommend to have? To help improve your experience?

  Users adherence:
- 3- What were some common reasons for dropout or unresponsiveness?
- 4- What helped in improving the adherence? (compensations divided into 3, etc...?)
- 5- How much do you believe that an electronic self-help service can be useful and accepted in Lebanon?

- Helper retention:
    - 6- Can you identify anything that may make helpers more likely to continue their employment as an e-helper?
    - 7- Do you think users will be able to user Step-by-Step without the e-helpers support at some point?
    - 8- Are there any changes required to improve the experience in this program? (adding more calls per week, longer calls? Chatting versus messaging?)
    -

- Integrating the role of supervisor into your workload:
    - 9- How do you view your role as clinical/admin supervisor to the e-mental health intervention considering your workload? Do you feel you need to allocate more or less time?
        - Explore barriers and facilitators to integrating the helping role into previous work.
    - 10- What is your overall impression on the supervision model (weekly meetings/face to face) how do you describe its effectiveness in solving problems encountered? (were you able to discuss and solve challenges in your team meetings?)
    - 11- Any other recommendations regarding the support of the e-helpers to solve the challenges? i.e. improving the way of supporting, the time per week, the individual discussions, ect.
    - 12- Any recommendation to improve the coordination process between all project team members?
- Training needs of helpers:
    - 13- Based upon your experience, what would you suggest we change about the way the training is delivered? (e.g. language of training, length, role plays, classroom set up?)
    - 14- Based on your experience, what would you add or change about the helper manual and training curriculum?
    - 15- what would you add or change about the helper manual and other materials (SOPs, etc..)? is there anything that you wish was there? More guides, tips, processes, templates?
    - 16- Based on your experience, how was the orientation phase including training and support provided during the initiation phase? Was the training length and structure acceptable? (3 days theory about research and intervention and 3 half days role plays).

Do you recommend any changes? is it sufficient and clear to start the implementation phase directly or is there a need for further training? (Would you suggest any changes?)

- 17- To what extent do you feel the e-helpers implemented the skills that they were trained in and that you taught them after they started their roles?
  - Explore barriers and facilitators to skills development
- 18- In your opinion, are the protocols suitable for all potential participants? What needs to be improved? (safety protocols etc…)

4. Review any written records with the interviewee still present. If anything is not clear ask for clarification and correct written notes as necessary.

5. Ask the interviewee if they have anything to add. Any additional information is added to the interview notes as required.

6. Thank person and leave.
