# Supplementary material for: Feasibility and uptake of a digital mental health intervention for depression among Lebanese and Syrian displaced people in Lebanon: a qualitative study
Source: Front Public Health. 2024 Jan 22;11:1293187. doi: 10.3389/fpubh.2023.1293187 (PMC10840138; doi:10.3389/fpubh.2023.1293187)
Supplement: Supplementary material 4 — Interview guide with study participants. [file Data_Sheet_4.pdf]

Interview guide with study participants- (online)

Increasing Access to Mental Health Care for People in Lebanon Living in Adversity: "Step-by-Step" intervention

Greet person. Introduce self, including what organization you are working for. Explain the study following written informed consent process (Consent form at the end of this document).
Possible additional explanation of semi-structured interview process:
We would like to ask you some questions about your experience in Step-by-Step, to help us to think about how it could be improved in the future. There are no right or wrong answers to the questions we are going to ask. The interview will be recorded without any identifier to make sure that answers are transcribed accurately; all answers will be reported anonymously to ensure confidentiality. We will be speaking to a number of people, asking everyone the same questions. If you feel unable to answer a question please say and we will move on to the next one.

In note book document date and site of interview, age and gender of interviewee, their position but not their name (i.e. intervention participant, e-helper), and initials of interviewers.

Begin semi-structured interview:
Record responses and make pertinent notes in the notebook.
Interview process:

- Overall impressions:
    - *1- Please describe your experience in Step-by-Step*
        - Explore positive / negative views through probes.
        - To what extent did it meet your expectations? What were you hoping to get out of it?
        - How was your experience in being part of a research study?
        - How was your experience in being part of electronic self-help program?
        - What did you find good or not so good about it?
        - Have you experienced any technical issues or other issues using SbS? If yes get a short description using the probes:
        - Can you describe to me the problem overall.
        - Now if you can give a bit more details
- Intervention:
    - 2- Can you tell me a bit about how often you used SbS and how many sessions you completed? What was your overall impression of it?
    - 3- Ask the participant to think back to their experience of using the website/app and ask about experience using the app, including features and design. (how was it for you to navigate through the sessions, exercises, did you like the colors, etc..)
        - What are the features/exercises you used and liked the most?
        - What are the features/exercises you didn't use and why?
        - Are you using / have you used any of the learnings from SbS still now?
            - If yes, what are you using?
            - If no, what's got in the way?

Interview guide with study participants- (online)

- Did you download the audios to listen to the story or did you prefer to read it/calendar?
- Could ask this in a more open way. Was there any point in the intervnetion that you remember the exercises being more complicated or too difficult, or *less helpful?*
- What about the challenging activies?
- Could we also ask about the mood tracking? E.g.:
  What do you think about the mood tracker? Did you use it? How often / when did you use it?

- What alternative features or options do you recommend to have? To help improve your experience?
  - Probe: is there anything you would add? Anything you would remove?

- 4- What did you think of the story? (probe if needed: how did you feel about its relevance, redundancy, difficulty, engaging and captivating...)
  - Was there any preferred session? Or a session you didn't like? Why?
- 5- What did you think of the audio exercises? (probe if needed: grounding and relaxation; idea, difficulty, effectiveness voice, pace, placement in the app)
  - What could be improved?
- 6- What did you think of the interactive activities?
  - What could be improved?
  - What do you think about the logos and icons? Are they meaningful?
  - What are some coping mechanisms you are using in the current situation? (before Covid, after Covid and after explosion) And are the examples relevant to the current stressors? (covid, explosion)
- 7- How did you use the info/list in the program, in your daily life?
- 8- Would you recommend others to use the app? Why?

- Rapport with helper:
  - 9- Please describe how you found working with your helper
    - Explore positive / negative views through probes. (technical, motivational, emotional)

  - 10- How did your family view calls you received or your relationship with your helper?
    Explore positive / negative views through probes.

  - 11- How could we improve the contact methods, for example the timings or how we contact users?
    - Explore further if they are not clear.
    - Number of calls per week/ duration of calls
  - 12- Do you think the app would function well without the e-helpers? Why?
- Intervention adherence:
  - 13- Please describe how easy or difficult you found it to complete the five sessions
    - Explore barriers and facilitators to attendance.

    - Frequency of sessions, length, video vs slides

- 14- Please describe how you found implementing the skills you learned in the programme in your everyday routine
    - Explore barriers and facilitators to skills development.

- 15- Is there anything else we could do to help users stay motivated?
    - Explore further if they are not clear.

- 16- How could we encourage users to use SbS as intended e.g. reading the story, completing the interactive parts, making use of the weekly support? How could we encourage users to keep the app and fill the assessments two and five months later?

  17- How we can make it easier for people to use the techniques in their daily life or come back for more guidance to the program?
    - What can be done to help users benefit from the app (videos, reminders, interactions)

- Registration/assessments:
    - 18- Ask the participant to think back to their experience registering (how was it for you to navigate through the questionnaires, study information, consent, did you like the colors, etc..)
    - 19- How much information would you want to receive about the service in the future? and how?
    - 20- Please describe how easy or difficult you found doing all the assessments, consenting and registration for this research

Review any written records with the interviewee still present. If anything is not clear ask for clarification and correct written notes as necessary.

Ask the interviewee if they have anything to add. Any additional information is added to the interview notes as required.

Thank person and end the interview
