# Supplementary material for: Feasibility and uptake of a digital mental health intervention for depression among Lebanese and Syrian displaced people in Lebanon: a qualitative study
Source: Front Public Health. 2024 Jan 22;11:1293187. doi: 10.3389/fpubh.2023.1293187 (PMC10840138; doi:10.3389/fpubh.2023.1293187)
Supplement: Supplementary material 5 — Table 2. recommendations. [file Data_Sheet_5.pdf]

Table 2. Suggestions and recommendations by all key-informants pertaining to different implementation aspects of the project including content, features, support, communication, and sustainability

Domain	Recommendation
**Content-related**	Continuously investigate the reasons for dropout and implement measures to improve adherence.<br><br>Prepare tutorial animated videos on how to sign up and navigate through the app instead of texts alone.<br><br>Continuously make small adaptations and integrate messages to the content, to adapt to local situation changes (Covid-19; economic crisis, trauma post-explosions, etc…).<br><br>Show more examples on how to implement activities (ex: give more examples on the problems and solutions exercise).<br><br>Add a brief introduction and explanation of the benefits of each exercise in the app.<br><br>Add trailers about the next session with a teaser about the story events to help keep the users intrigued and engaged until the next session opens.<br><br>Make changes to the stories or add new ones to make sure they attend to a wider audience such as migrant workers, workplace-related scenarios, and others.<br>➢ adapt the story to the refugees' struggles by adding hints about the deteriorating quality of life and the homelessness that is increasingly encountered.<br>➢ add parenting tips related to dealing with teenagers.<br>➢ add a session on relationship problems and exercises to boost self-confidence.
**App-related features**	Continuously update and upgrade the app to ensure it is not outdated. Always benchmark best practices for retaining users and learn from the private market/apps about engaging methods<br><br>Add notifications like *"we haven't seen you in a while", "don't forget to visit us".*<br><br>Add helpful daily tips to motivate users to commit to practice, for example *"Did you know that if you practice this everyday, you're increasing your endorphins and you feel better?"* or link it to the current situation *"During COVID19 you can do this and that".*<br><br>Amend the interactive part of the app to make it more user-friendly, example: have a drop-down list (fast access) for all options of activities, simplify the layout of the online calendar and make it accessible from the start so that people learn how to use it for all activities.<br><br>Show mood tracker results to the e-helpers to better help them track the progress of the users.<br><br>Upgrade the admin platform to facilitate the work of e-helpers.<br><br>Downsize the app and attend to the slow internet problems in Lebanon which might be the biggest hindering factors to the uptake of Step-by-Step.<br><br>Explore different outlets and methodologies to deliver SBS to people with intellectual disabilities or impaired vision.

	Have at least one IT professional based in Lebanon to address any bugs or changes in a timely manner.<br><br>Establish a direct feedback mechanism in the app to receive comments from the users about the service received.
**Assessments**	Reduce the number of assessments or divide them into different parts to be filled at different points in time, and to make them less overwhelming.<br><br>Explain and clarify medical concepts such as "panic attacks".<br><br>Explain certain technical terminologies and add the English or French translation when needed because the local population might understand them better in different languages, ex: psychologist vs psychiatrist
**E-helpers' support protocols, and follow-up measures**	Refine protocols to account for implementation changes and potential risks encountered and update the support templates accordingly.<br><br>Conduct an introductory call with all participants to explain the project, manage expectations, and set an oral contract about the users' commitment:<br><br>➢ clarify the role and scope of the e-helpers' support, and the duration of the calls.<br>➢ emphasize the self-help component so that users know that they need to be proactive and commit to the program.<br>➢ set a limit for the number of calls they receive (8 weeks max) to avoid dragging in the intervention for many months.<br><br>Contact users only after they have completed a session to have a meaningful support session and avoid inefficient reminder calls.<br><br>Set an outline for calls and mention it at the beginning of each session and keep track of time.<br><br>Create a list of WhatsApp FAQs with message templates to effectively respond to frequent questions.<br><br>Improve the app by adding more automated reminders, clear explanations, and phone support to help Syrians better understand and participate in exercises and activities.<br><br>Adopt WhatsApp as a means for follow up with users who don't respond on the platform after receiving their consent.<br><br>Establish agreements with partnering service providers such as health facilities or hospitals and convey the high-risk protocols to them, to ensure a smooth and timely response upon referral.
**E-helpers' training**	Update the training material and modality:<br><br>➢ extend the training for a few more days.<br>➢ split the theoretical part and role plays into two trainings, with a gap period of one week between the theoretical and practical to familiarize the team with the manual and content.<br>➢ train e-helpers on responding to local and international crises such as the COVID-19, the Beirut Blast, the surge of SGBV cases.<br>➢ conduct a training for case notes taking and unify template.

	➢ plan a refresher session to make sure there are no confusions in the process and that the quality of care is still maintained.
**Supervision**	Refine the supervision forms to better reflect the support given during the sessions.<br>Conduct individual supervision sessions occasionally to ensure tailored and personalized feedback for each e-helper.<br>Devise an assessment and improvement plan for each e-helper.<br>Conduct self-care trainings, one on one sessions, and create a mental health promoting environment at work to avoid burnout and compassion fatigue among e-helpers.
**Communication about SbS and integration into the system**	Invest in dissemination and promotion activities about SbS to ensure roll out and uptake in the community. Conduct national awareness campaign about SbS through mass and social media and outreach.<br>Segregate the population into different demographic groups: target young adults and tech savvy people through social media, mainly Facebook and Youtube, and older adults and less tech savvy people through mass media by hosting TV morning and evening shows, TV news, and commercials.<br>MoPH to send SMS and app notifications about SBS, just like the ones for COVID19, to reach the widest pool of Lebanese.<br>NGOs working on NCDs to advertise for SbS as evidence suggests that there is high possibility of comorbidities between NCDs and mental health disorders.<br>Disseminate SBS through the Mental Health and Psychosocial Support Taskforce (MHPSS) by hanging promotional material and sharing information in peer sessions via the outreach volunteers or case workers.<br>NGOs working with Syrian displaced population and Palestinians should be targeted as well, and UN agencies such as UNHCR, UNRWA, UNICEF and their partners.<br>Clearly emphasize throughout all communications that the app is private and confidential, as this would encourage people to sign up for it.<br>Feature testimonies (real people instead of celebrities) of success stories from previous users would increase the credibility massively. Users can reveal a sneak peek of the content of the app.<br>Publish the results of the research phases in scientific journals for global dissemination and for attracting different donors.<br>Launch a separate Facebook page for SbS to be able to boost it and track the traffic on it.<br>Clear messaging needs to differentiate between SbS and the National Hotline for Emotional Support and Suicide Prevention to avoid confusion between the two services.
**Sustainability**	Develop a sustainable integration plan into the current health care services is needed, to ensure continuous referral to SbS. Outreach volunteers and NGOs would play a crucial role in the roll-out of this intervention in the community.

	Insurance companies can consider including this app into their financial coverage. This will help advertise for it and also sustain part of its operations on the long run.
